# Brief hospitalizations of elderly patients: a retrospective, observational study

**DOI:** 10.1186/1757-7241-22-17

**Published:** 2014-03-07

**Authors:** Sofie Strømgaard, Søren Wistisen Rasmussen, Thomas Andersen Schmidt

**Affiliations:** 1 The Emergency Department, Holbæk University Hospital, Holbæk, Denmark

**Keywords:** Length of stay, Elderly, Old, Inappropriate hospitalization, Emergency department

## Abstract

**Background:**

Crowded departments are a common problem in Danish hospitals, especially in departments of internal medicine, where a large proportion of the patients are elderly. We therefore chose to investigate the number and character of hospitalizations of elderly patients with a duration of less than 24 hours, as such short admissions could indicate that the patients had not been severely ill and that it might have been possible in these cases to avoid hospitalization.

**Methods:**

Medical records were examined to determine the number of patients aged 75 or more who passed through the emergency department over a period of two months, and the proportion of those patients who were discharged after less than 24 hours. The reasons for the hospitalization, the diagnoses and the treatment given were noted.

**Results:**

There was a total of 595 hospitalizations of patients aged 75 or above in the emergency department during the period. Twenty-four percent of the older patients were discharged after less than 24 hours. Of these, 40% were discharged from the emergency department. The most common problems leading to hospitalization were change in contact or level of consciousness, focal neurological change, red, swollen or painful leg conditions, dyspnea, suspected parenchyma surgical disease and problems with the urinary system or catheters. The most common diagnoses given at hospital were chronic cardiovascular disease, bacterial infection, symptoms deriving from bone, muscle or connective tissue, liquid or electrolyte derangement and observation for suspected stroke or transient cerebral ischemia. Eight percent of the patients required telemetry, 27% received intravenous liquids, 30% had diagnostic radiology procedures performed and 3% needed invasive procedures. Other types of treatment given included electrocardiography, laboratory examinations, oxygen supplements, urinary catheterization and medicine administered orally, subcutaneously, as an intramuscular injection or as an inhalation.

**Conclusion:**

There appears to be a group of patients who cannot be adequately handled with the resources of the primary health care sector, yet who do not belong at the emergency department. Further studies are needed to create a suitable service for these patients, and to improve the continuity of the treatment and the cooperation between hospitals and the primary health care sector.

## Background

Most of the patients in Danish hospitals, especially in the medical departments, are elderly. Demographic studies suggest that in the future, a greater proportion of the population in Denmark will be elderly, thus we expect a larger amount of elderly patients [1]. Potentially preventable hospitalizations for acute conditions are seen more frequently in older patients than in younger [2].

Hospitalization of old persons is often followed by a decline in function beyond what can be explained by the disease for which the patient was admitted. Immobilization, high bed rails, inaccessibility of fluids, isolation, lost glasses and hearing aid and accelerated bone loss caused by bed rest are problems associated with hospitalization, that may lead to deconditioning, fall, syncope, dizziness, fracture, delirium, dehydration, malnutrition, pressure sore and infection [3,4]. Gilick et al. have found that the risk of developing confusion, not eating, incontinence and falling during hospital admission, where the problem could not be explained by the disease for which the patient was admitted, was significantly higher in older patients than in younger [5]. Other studies have shown that a loss of ADL (Activities of Daily Life) functions often occur during and after a hospitalization of elderly, especially in the oldest patients, patients with low gait speed and those with lower functional or mental status scores on admission [6,7]. However it is not possible to conclude from those studies how much of the decline in functional status that could be attributed to the hospitalization itself and how much was caused by the disease for which the patients were hospitalized. A study by Covinsky et al. found that in some elderly patients, decline in ADL function was seen in the weeks before, during or after hospitalization. The risk of functional decline was increasing with the patient’s age. The risk associated with increasing age was smaller for pre-hospitalization decline than for new decline in in the hospital or failure to recover in the hospital. After adjusting for comorbidity, illness severity and other confounders, it was only during hospitalization that the risk of functional decline was increasing with age [8].

Furthermore, old patients who are hospitalized often experience a prolonged stay in the emergency department which increases the risk of in-hospital adverse events and a longer hospital length of stay [9].

Since hospitalization may be harmful for old patients it is relevant to study the hospital admissions of the elderly patients in order to improve the courses and to avoid admissions when possible. In this study we chose to examine the hospital admissions of less than 24 hour duration with the assumption that if a patient can be discharged less than a day after hospitalization, this indicates that the patient might not have been severely ill. In those cases one could consider if the problem that caused the hospital admission could have been solved in a more appropriate way without hospitalization.

The purpose of this study was to examine the amount patients aged 75 years or above admitted through the emergency department with a hospitalization period of less than 24 hours and to study the contents of these hospitalizations: the reason for hospitalization, the diagnoses and the treatment given at the hospital.

## Methods

This was a retrospective study where the dossiers of patients admitted to the emergency department of Holbæk Hospital during September and October of 2010 were reviewed. The dossiers of patients older than 75 years were studied in order to find the proportion of elderly patients discharged after less than 24 hours. This was defined as more than 24 hours between the time noted in the dossier at the time of hospitalization and the time noted in the dossier at the time of discharge. Institutional approval was obtained to perform the study.

Hospitalizations where the patient was transferred to a somatic department at another hospital after less than 24 hours but in total was hospitalized for more than 24 hours, were counted in the group of long hospitalizations. Cases where the patient was moved to palliative or psychiatric departments after less than 24 hours were counted in the group of short hospitalizations. The cause of hospitalization according to the part of the dossier made at the time of hospitalization and the diagnoses given at the end of hospitalization were noted. Some of the patients had more than one problem or symptom leading to hospitalization and some patients were given more than one diagnosis at the time of discharge. In those cases we noted what we perceived as the main problem. If a patient was admitted more than one time during the period, each hospitalization was counted separately. The treatment given and procedures performed during hospitalization were divided into different categories as shown in Table 1.

**Table 1:** Proportion of patients receiving different types of treatment

**Treatment or procedure**	**Number of patients**	**Proportion of patients**
Laboratory examinations such as blood samples, urine stick test and cultivation	96	67.6%
Electrocardiography	64	45.1%
Diagnostic radiology procedures	42	29.6%
Medicine administered per orally, suppository or as an ointment	41	28.9%
Intravenous medicine or liquid	38	26.8%
Urinary catheterization	16	11.3%
Telemetry	11	7.7%
None	10	7.1%
Oxygen supplement	6	4.2%
Medicine administered subcutaneously, intramuscular or as an inhalation	6	4.2%
Other	5	3.5%
Invasive procedures	4	2.8%

The left column shown the different types of treatment or procedures performed during the short hospitalizations. The following columns show the number of patients and the percentage of patients receiving each type of treatment (n = 142). Note that there is an overlap since some patients receive more than type of treatment.

### Statistics

Results are given as number of observations and proportion or percentage of observations.

## Results

The general characteristics of the study sample are described in Table 2. There was a total of 595 hospitalizations of patients aged 75 years or above in the emergency department. Of these, 451 hospitalizations were of more than 24 hour durance, 142 lasted less than 24 hours, 2 ended within 24 hours because the patient died and 2 because the patient left the hospital without having been examined by a physician. No patients were discharged directly from the emergency department to psychiatric or palliative care. One patient was discharged from the department of internal medicine to psychiatric care after less than 24 hours. Two patients were discharged to palliative care after less than 24 hours admission internal medicine. Among the patients who were initially hospitalized for less than 24 hours, 26 patients were readmitted during the study period.

**Table 2:** General demographic characteristics of the study population

	**Men**	**Women**	**Total**
Number of patients	279	279	558
Mean age	82,25	83,72	82,99
Median age	82	83	83
Range	75-96	75-99	75-99

The hospitalizations where patients left the department without being seen are excluded from further analyses. In the hospitalizations lasting less than 24 hours, in 57 cases the patients were discharged directly from the emergency department and in 85 of the admissions the patients were referred to other departments at the hospital. I.e. 24% of the admissions lasted less than 24 hours and of these 40% were ended in the emergency department. In the group of hospitalizations where the patients were admitted to other departments at the hospital for less than 24 hours, 67% were directed to the a medical department, 5% to the department of general surgery, 7% to the department of orthopedic surgery and 21% to the urological department.

In the hospitalizations lasting longer than 24 hours, 69% of the patients were admitted at the medical department, 13% at the department of orthopedic surgery, 15% at other surgical departments and 3% at the intensive care unit.

**Figure 1:**
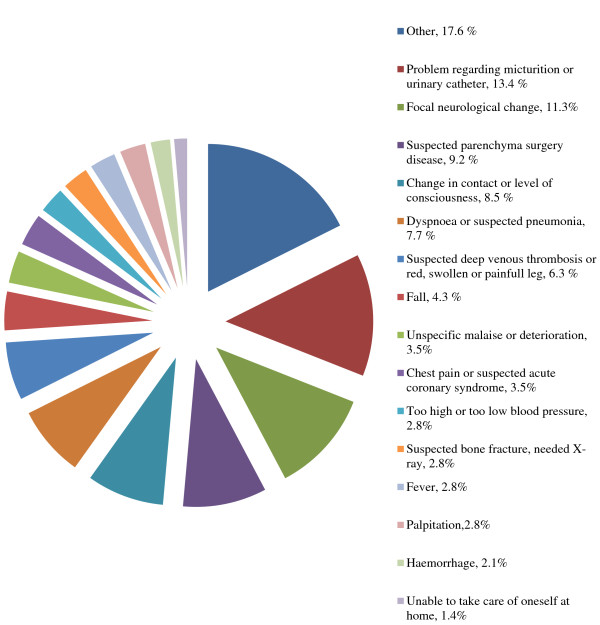
**Causes for hospitalization (n = 142).** The problems leading to short hospitalizations. The most common were urinary problems, focal neurological change, suspected parenchyma surgery disease, change in contact and dyspnea.

**Figure 2:**
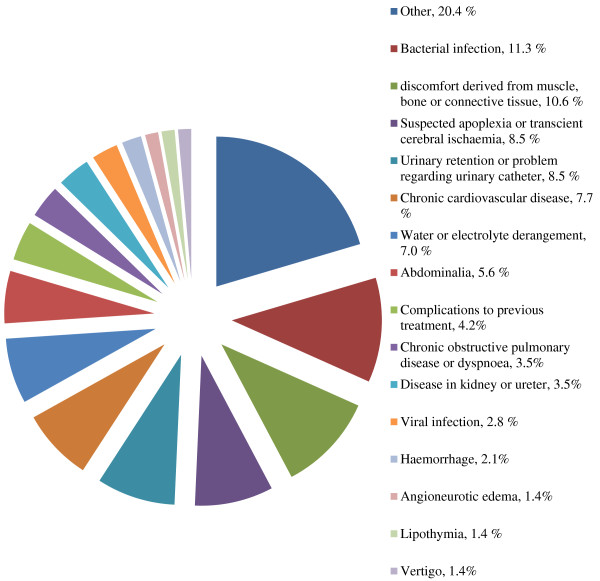
**Diagnoses in short hospitalizations (n = 142).** The diagnoses noted in the discharge letter at the end of hospitalization. The most common were infections, discomfort from muscle, bone and connective tissue, suspected apoplexy and urinary problems.

Table 1 shows how many patients received the different types of procedures and treatments during the short hospitalizations. Table 3 shows the treatment given for those who were actually admitted and those who were discharged directly from the emergency department. Figure 1 shows the proportion of the different causes of short hospitalizations. There was a great variation in the causes of hospital admission. Some common causes were suspected deep venous thrombosis, change in contact or level of consciousness, focal neurological change, suspected parenchyma surgery disease, dyspnea or problem with urinary catheter. In Figure 2, the diagnoses that the patients received at the end of the short hospitalizations are divided into different categories. The most common diagnoses given were chronic cardiovascular disease, bacterial infection, symptoms derived from muscle, bone or connective tissue, liquid or electrolyte derangement and observation because of suspected apoplexy or transient cerebral ischemia.

**Table 3:** Actions performed for those who were actually hospitalized for less than 24 hours and for those who were discharged straight from the emergency department

**Treatment or procedure**	**Hospitalized from emergency department to other department for less than 24 hours, n = 85**	**Discharged directly from emergency department, n = 57**
Laboratory examinations such as blood samples, urine stick test and cultivation	73 (85.9%)	23 (40.4%)
Electrocardiography	46 (54.1%)	18 (31.6%)
Diagnostic radiology procedures	33 (38.1%)	9 (15.8%)
Medicine administered per orally, suppository or as an ointment	29 (34.1%)	12 (21.1%)
Intravenous medicine or liquid	33 (38.1%)	5 (8.8%)
Urinary catheterization	11 (12.9%)	5 (8.8%)
Telemetry	11 (12.9%)	0 (0%)
None	0 (0%)	10 (17.5%)
Oxygen supplement	4 (4.7%)	2 (3.5%)
Medicine administered subcutaneously, intramuscular or as an inhalation	6 (7.1%)	0 (0%)
Other	5 (5.9%)	0 (0%)
Invasive procedures	4 (4.7%)	0 (0%)

## Discussion

In conclusion, in this study 24% of the patients admitted to the emergency department were discharged after less than 24 hours. Of these, 40% were discharged directly from the emergency department. There was a great variety in the problems leading to the hospitalization and the diagnoses given at the time of discharge. It is worth noticing, that about 9% of the patients were admitted for a change in contact or level of consciousness. These patients might have delirium and might be particularly vulnerable to the potential harms of hospitalization. It might be relevant for future studies to look specifically at this type of patients.

Previous studies have examined ways to reduce inappropriate hospitalizations of elderly. Cherubini et al. have found that among Italian nursing home residents, those hospitalized had significantly fewer nursing assistant hours per resident per week than those not hospitalized [10]. However, a review of the literature from North America shows contradictory results of nursing staff on hospitalization from nursing homes. Evidence is unclear regarding the effect on hospitalization rates of nursing home’s provision of services such as intravenous therapy, x ray and laboratory.

In the United States, increasing the economic incentive for the physician to see the patient at the nursing home resulted in lower hospitalization rates, comparable quality of care and cost saving. Several studies have shown that nursing home based hospices reduce unnecessary hospitalization [4]. However as the review included only studies from North America, the results might not apply to European health care systems.

Another review suggests that the use of home health for post-acute care can be a cost-effective intervention to reduce readmissions for chronic conditions, but the effect has been studied in a limited set of conditions and populations. A study of COPD (chronic obstructive pulmonary disease) patients showed that quarterly home visits by skilled respiratory nurses and physicians, monthly phone follow up and facilitated access to hospital resources without inpatient admission lead to lower hospitalization rates and decreased length of stay. It was unclear whether the benefits of enhancing home health care through increased nurse training and patient education is effective across conditions and which components are the essential ones. Another study showed no effect of community nurse visits. Randomized controlled trials have found that the use of a pharmacist transition coordinator to enhance medication management reduces the odds of readmission. Influenza vaccination has also been shown to reduce hospitalization from nursing home. Improving home care through the use of screening tool to assess mental status, health status and social support reduced hospitalizations by identifying health problems. In both nursing home and home health settings, increased monitoring, assessment and the use of data appear to reduce hospitalizations [11].

A possible way to reduce hospitalizations of elderly or reduce the length of hospitalization may be short stay units or observation units in the emergency departments for patients with expected short length of stay. A systematic review found weak evidence that observation units reduce the length of stay and reduce admissions [12]. It has been shown that short stay units improve patient satisfaction and quality of life [13,14].

“Hospital at home”, where the patient stays in his or her home and receives the treatment that otherwise would have been given at the hospital, may be a good alternative to hospital admission for some elderly patients. A study of “hospital at home” versus traditional acute care hospital admission for old patients with COPD, chronic heart failure, pneumonia or cellulitis found that patients in “hospital at home” on average improved their ADL function during the treatment while patients in the acute care hospital in average deteriorated in ADL function even though they received more formal physical or occupational education. However, the study was nonrandomized and the patients in the “hospital at home” group had a lower level of function prior to admission which may put a ceiling effect on the possibility to decline further in ADL function [15]. A randomized study of “hospital at home” versus acute care hospital for old patients with COPD showed no difference in ADL function between the two groups but there were significantly fewer readmissions, longer time between discharge and readmission and greater improvements in depression and quality of life scores in the “hospital at home” group [16].

This is a preliminary investigation and limited conclusions can be drawn. It was a retrospective study which in general does not provide as good evidence as prospective studies. However since it was not an interventional study we consider this circumstance unlikely to have impact on the results. The study was performed only in 2 months of the year so it does not take into account that there might be a seasonal variation in the problems causing hospitalization of elderly patients. For example one could imagine more dehydrated patients in the summer or more patients with femoral fractures in the winter. Furthermore, we studied the short hospitalizations only at Holbæk University Hospital, not at other hospitals in the region. There might be differences in the type of patients at the different hospitals in the region because the hospitals do not have the same type of departments. For example not all hospitals have a department of urology. If the patient is suspected to have a urologic disease, he or she will be sent to a hospital with a urological department, so there might be differences in the quantity of the different types of patients at different hospitals.

Whether or not a hospitalization was unnecessary or appropriate is an occupational judgment and therefore to some extent subjective. We chose the criteria of less than 24 hours durance as an indicator because this gives an exact limit of which patients to count to the group of inappropriate hospitalizations and which not. There might be cases where most doctors would agree that the hospitalization was appropriate even though it lasted less than 24 hours. On the other hand, there can be situations where most physicians would consider the hospitalization unnecessary when reviewing and analyzing the patient course despite a durance of for example 25 hours, which would mean that it would not be counted in the group of short hospitalizations with our definition. Thus we did not make an individual professional judgment of the relevance of each hospitalization. However, as seen on Table 1, most of the procedures performed or treatments given were quite simple. There might be a health policy problem since most of these patients probably could have been discharged after a short management in the ED. These admissions might increase the hospital care costs. Further studies of the organization of health care services for the elderly are needed.

## Competing interests

The authors declare that there are no competing interests.

## Authors’ contributions

Sofie Strømgaard participated in the design of the study, collected data and drafted the manuscript. Thomas Andersen Schmidt and Søren Wistisen Rasmussen participated in the design of the study and helped draft the manuscript. All authors read and approved the final manuscript.
